# Current Trends on Green Wireless Sensor Networks

**DOI:** 10.3390/s21134281

**Published:** 2021-06-23

**Authors:** J. Carlos López-Ardao, Raúl F. Rodríguez-Rubio, Andrés Suárez-González, Miguel Rodríguez-Pérez, M. Estrella Sousa-Vieira

**Affiliations:** atlanTTic, Escola de Enxeñaría de Telecomunicación, Universidade de Vigo, 36310 Vigo, Spain; rrubio@det.uvigo.es (R.F.R.-R.); asuarez@det.uvigo.es (A.S.-G.); miguel@det.uvigo.es (M.R.-P.); estela@det.uvigo.es (M.E.S.-V.)

**Keywords:** Wireless Sensor Networks, energy harvesting, energy-efficient data communication, energy management, energy prediction

## Abstract

The issue of energy balancing in Wireless Sensor Networks is a pivotal one, crucial in their deployment. This problem can be subdivided in three areas: (i) energy conservation techniques, usually implying minimizing the cost of communication at the nodes since it is known that the radio is the biggest consumer of the available energy; (ii) energy-harvesting techniques, converting energy from not full-time available environmental sources and usually storing it; and (iii) energy transfer techniques, sharing energy resources from one node (either specialized or not) to another one. In this article, we survey the main contributions in these three areas and identify the main trending topics in recent research. A discussion and some future directions are also included.

## 1. Introduction

Wireless Sensor Networks (WSN) is one of the most active research areas with applications in numerous fields such as transport, health, military, agriculture, environment monitoring and control, etc. Within this research area, the problem of energy balancing in WSNs (harvesting, transfer and conservation) has been always the one that has attracted the most interest.

A WSN is a set of sensor nodes wirelessly interconnected with at least one central node, called base station (BS) or sink node. The base station can both control the network in a centralized way and communicate with end users or/and other networks.

A sensor node is an electronic device that essentially has four main components: a sensing unit, a processing unit, a communications unit and a power unit. Other equipment such as a mobility unit or a position tracking unit are optional. The sensing unit allows the node to collect data related to its ambient conditions. These data are handled by the processing unit, and its communication unit is used to exchange data with other nodes and the base station. The power unit provides the necessary energy to the node and it is typically a chemical battery.

A WSN may contain one or more BSs together with a lot of sensor nodes, static or mobile. Using many nodes enables the simultaneous acquisition of data related with the environment conditions in very wide areas. This makes WSNs ideal for an increasingly wide range of tasks such as fire detection, weather forecasting, energy management, biomedical applications, environmental and habitat monitoring, surveillance and reconnaissance, home automation, object tracking, traffic control, inventory control, agriculture, machinery failure diagnosis and various military applications.

In a wide sense, the objective of a WSN is to feed some application with data dynamically generated by sensor sources distributed in a certain region and, as a result of the analysis of such information, other devices may be commanded remotely for some action.

Usually, sensor nodes have a limited power source. Since in many cases the nodes are deployed in unreachable areas, it is not possible to recharge or replace the battery of the sensor nodes. Therefore, it is necessary to find appropriate techniques to save energy by reducing the power consumption of the nodes and so improving the lifetime of the WSN. It is widely recognized that sensors use more energy during communications than during sensing and preprocessing the data they must send. Thus, the radio is the main problem when it comes to extending the life of devices that depend on batteries to perform their mission and, therefore, the communications unit, which involves both transmission and reception of data, uses a significantly higher proportion of the available energy [1].

There are several aspects of a WSN dealing with sensible energy strategies: conservation, harvesting and transfer.

**Energy** **conservation** techniques simply aim to extend the lifetime of the network by reducing the energy used while the WSN continues to operate as required [2]. Energy saving usually implies minimizing the cost of communication at the nodes since it is known that the radio is the biggest consumer of the available energy [3]. Different energy-saving schemes were recently surveyed in [4,5].
**Energy-harvesting**  techniques seek to increase the energy available to the nodes. The energy can be obtained from the external environment such as solar, wind, vibrations, radio frequency, thermal, etc. These techniques convert energy from the environment into electrical energy for the nodes. In [6] it is presented a recent survey. A major constraint of these techniques is that energy sources are not always accessible and therefore it is necessary to store the harvested energy using rechargeable batteries or low-powered supercapacitors.
**Energy** **transfer** is another emerging technique applied to extend the lifetime of the network. The idea is that energy-rich nodes transfer energy to energy-deficient nodes. This transfer can be done wirelessly from a node specialized in energy harvesting or a node with enough energy resources to a node in need of energy in the same network. Engmann et al. [7] review several mechanisms for energy transfer such as inductive and magnetic coupling, or electromagnetic radiation.

Usually, many energy balance approaches involve using a combination of several schemes to address the efficient use of energy of the WSN [7].

This paper aims to survey the main contributions of these three types of techniques for extending the lifetime of WSNs (conservation, harvesting and energy transfer), also identifying the main research topics that are trending presently. There are many surveys addressing one of these three categories, but there are only a few surveys that covers all the types, one published in 2017 by Yetgin et al. [8] and another one published in 2018 by Engmann et al. [7]. In this survey, we will intend to classify and summarize the different techniques proposed in the literature, since at least 2018, for conservation, harvesting and transfer of energy in WSNs, focusing on the most recent surveys and, mainly, trying to identify the trending research topics and the most active areas at present, commenting the most relevant and interesting works in each case. The bibliographical revision was closed on November 2020.

The paper is organized as follows: After introducing the subject and the objectives of this survey, in Section 2 we present the mechanisms—and the latest research—that seek to extend the life of the sensor network by reducing the energy expenditure associated with some of the usual processes in which a sensor is immersed. In Section 3 we address the issue of energy harvesting using natural energy sources. In Section 4 we review the work that would allow the use of radio frequency signals for the transfer of energy between network elements. There is a discussion and hints for future directions in Section 5. Finally, in Section 6, we present the conclusions.

## 2. Energy Conservation

Energy conservation methods try to reduce energy consumption of the sensor nodes to extend the lifetime of the nodes and, therefore, the lifetime of the network. Since radio communication is the main consumer of energy in a node, the design of communications protocols (MAC and routing software) is a key element. In the past, without rechargeable batteries, prolonging the life of the network was the major focus in the communication unit design, leaving network latency and throughput optimization at a second place. Since energy-harvesting capabilities has been added to nodes, Quality of Service (QoS) has come back to be its main focus, though energy constraints remain. Energy-Neutral Operation (ENO) arises as a new paradigm, and achieving the best performance of the application/network (in terms of throughput and time response) balancing the energy expenditure caused by sensor activity with the energy flow being able to harvest has attached attention in recent times.

There is abundant literature relating to energy-saving in WSNs in recent years that have been collected and classified in different works. The survey in [9] uses a simple classification based on the network structure (flat or hierarchical) for data aggregation and routing schemes. This year two surveys have been published. In [4] energy-saving schemes such as duty-cycling, efficient routing, efficient MAC, data aggregation, cross-layer design and error control code are addressed. This survey includes works until 2019 and it is noticeable that the most active year was 2017.

**Figure 1:**
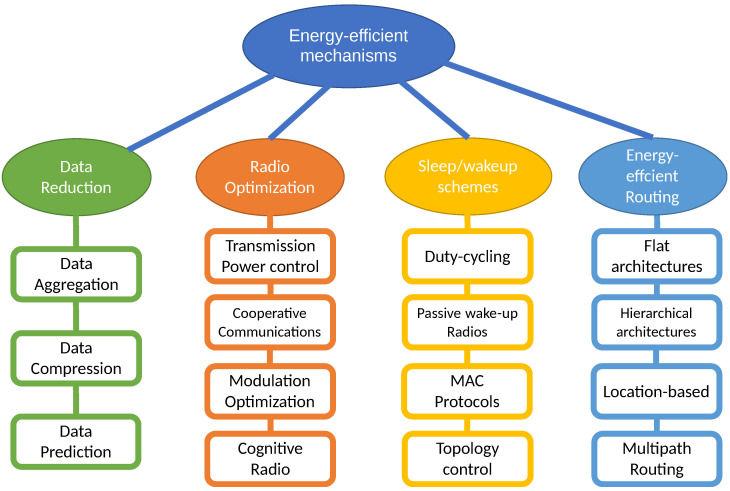
Taxonomy of energy-efficient mechanisms proposed in [10].

Another recent review by Singh et al. [5] uses an uncommon classification, considering battery management schemes (nodal management and energy balancing), transmission power-management schemes (MAC protocols, routing protocols and transmission policy), system power-management schemes (processor power and device management) and other miscellaneous schemes like load balancing, duty-cycling or cross-layer optimization. In this survey we decide to use the four main categories proposed in [10] (see Figure 1). In every category we explain the fundamentals of each type of solution, we consider some subcategories and when we find a particularly active area of research in the last two years, we review the most relevant works found in the literature.

### 2.1. Radio Optimization

Since the radio module is the main component that causes the node battery to be exhausted, many researchers have addressed several mechanisms in wireless communications such as optimizing modulation or coding, cooperative communications, power transmission control, directional antennas or cognitive radio.

#### 2.1.1. Modulation Optimization

The goal of modulation optimization is to find the optimal modulation parameters to minimize the energy consumed by the radio. Existing research studies try to find a good trade-off between the information rate, the transmission delay, the constellation size, the distance between the nodes and the signal-to-noise ratio. Cui et al. [11] showed that to minimize the total energy consumption, for a given BER and delay requirement, the transmission time needs to be optimized, showing that it is possible to achieve up to 80% energy savings. The literature provides some evidence that low-order modulations such as BPSK are suboptimal for short transmission distances. In [12] it is shown that each modulation scheme has a single optimal SNR at which the energy consumption is minimized. The optimal SNR and the minimal energy consumption are larger for higher values of BER. Therefore, they found that operated at its optimal SNR, BPSK and QPSK are the optimal choices for long transmission distances, but as the transmission distance shortens, the optimal modulation size grows to 16-QAM and even to 64-QAM.

In recent years little interest has been shown in this research topic and only the work in [13] is noteworthy, where DMS (Dynamic Modulation Scaling), a technique which manages the constellation size to change the transmission time and energy consumption, is used in combination with topology control (see Section 2.3.4).

#### 2.1.2. Cooperative Communications

Cooperative communications strategies aim to enhance the quality of the received signal by operating several single antennas that work together to form a virtual multi-antenna transmitter, taking advantage of the fact that data are often overheard by neighboring nodes because of the broadcast nature of wireless networks, therefore gaining the benefits of multiple-input multiple-output (MIMO) systems while overcoming their challenges [14,15].

On the other hand, although a MIMO scheme is an effective way for reducing energy consumption in WSNs, the physical size and the energy available for the sensor nodes is limited, and therefore, installing multiple antennas at the sensor nodes is not ideal. Thus, cooperative MIMO (CMIMO) can be achieved by orchestrating collaboration among the single antennas installed on each sensor node. Therefore, spatial multiplexing can enhance the data rates, whereas spatial diversity can improve the bit-error rate (BER) using the CMIMO technique. Moreover, CMIMO minimizes the energy consumed in transmission, especially in long range transmission [16].

CMIMO is an area where recent research activity can be found in recent years. In [17], a novel protocol is proposed in which mobile terminals form a virtual MIMO uplink by means of device relaying on Device to Device (D2D) tier in 5G Cellular Networks. Its focus is to design an incentive for terminals to form the virtual MIMO and cooperate in relaying other data.

The work in [18] addresses how to extend the average battery capacity among the whole network through the selection of the cooperative coalition for CMIMO, proposing to apply the quantum-inspired particle swarm optimization (QPSO) algorithm to select the optimum cooperative coalitions of each hop in the routing path.

CMIMO scheme reduces the energy consumption of sensor nodes quite effectively by using the space-time block coding scheme. However, in [19] it is shown that in networks with high node density, the scheme is ineffective due to the high degree of correlated data. Therefore, to enhance the energy efficiency in these cases, the authors propose to use the distributed source coding (DSC) with the virtual MIMO data transmission technique. The DSC-MIMO first compresses redundant source data using the DSC and then sends it to a virtual MIMO link. The results reveal that energy consumption is lower than that in the CMIMO technique.

Aside from the pure CMIMO technique, CMIMO with spatial modulation (CMIMO-SM) [20,21] is another transmission approach recently proposed to achieve the spatial diversity of MIMO systems. The basic idea of CMIMO-SM is to give each individual antenna a preassigned index and let them cooperate with each other in a cluster to form a cooperative frame.

In [22], applications of CMIMO are proposed in networks of intelligent transportation systems (ITS) for reducing the total energy consumption. The scheme proposed is based on CMIMO-SM and its detailed energy consumption is compared with the traditional single-input-single-output (SISO) scheme.

#### 2.1.3. Transmission Power Control

Transmission Power Control (TPC) has been proposed in the literature to improve energy efficiency by adjusting the transmission power of the radio [23]. However, the TPC mechanism has an effect not only on energy but also on delays, interference and connectivity. In fact, when the transmission power is reduced, the risk of interference also diminishes. Conversely, the delay increases, because more hops will be needed to forward a packet. TPC has also been an active research area in recent years, essentially in the scope of Wireless Body Area Networks (WBANs) with medical applications, as shown in these two recent surveys [24,25]. Important work in this area is due to Sohdro et al. [26,27,28].

The aim of TPC mechanisms in WBANs, which can be combined with other energy-saving mechanisms [29,30,31], is to reduce the energy consumption and external interferences by dynamically adjusting the transmission power output, with the minimum effect on other performance aspects, such as reliability and latency. Other recent works in this field are adaptive algorithms based on variations in body conditions [32] or on human motion [33].

More recently, Qolami et al. [34] investigated TPC in 802.15.4 + RPL WSNs and proposed a mechanism where each node dynamically adjusts its transmission power based on channel conditions before sending every data and ACK packet. Their results confirm that this power control method improves network lifetime. A TPC mechanism is also proposed for Underwater Wireless Sensor Networks (UWSNs) in [35], where source nodes adjust transmission power according to the location of the destination node.

#### 2.1.4. Directional Antennas

Directional antennas are used to allow signals to be sent and received in more than one direction simultaneously, thus enhancing transmission range and throughput. Directional antennas may require positioning techniques to be oriented, but multiple communications can take place in close proximity, which results in spatial reuse of bandwidth. Many researchers have proposed the use of directional antennas to improve the energy efficiency of the network [36].

Given that omnidirectional antennas transmit a signal equally to all directions and, in most of the applications, communication is unicast, we can infer that significant amount of energy can be saved using directional antennas. In [37] the authors study the effect of directional antennas on energy efficiency of the IEEE 802.15.4 standard in outdoor WSNs. In [38] we can read a review of energy efficiency using directional antennas in mobile ad hoc networks and [39] is a very recent review of directional antennas for WSN applications, where a little section is dedicated to contention reduction and energy efficiency.

The use of directional antennas can also reduce the interference between radio streams and improve the SNR. Therefore, directional antennas can improve the reliability and reduce the number of retransmissions. However, use of directional antennas necessitates to find the right direction and parameters correctly and quickly. A recent work in this area [40] presents an energy-efficient localization of sensor nodes in WSNs using beacons from a rotating directional antenna.

Finally, we must note that the most active research area in recent years in order to take advantage of the properties of directional antennas is the proposal of new MAC protocols. The work in [41] contains a review of this topic but there are interesting subsequent works [42,43,44,45,46,47].

#### 2.1.5. Energy-Efficient Cognitive Radio

A cognitive radio (CR) is an intelligent radio that can dynamically choose a channel in the wireless spectrum and can set its transmission and reception parameters according to the channel status. The subjacent Software-Defined Radio (SDR) technology is intended to build fully reconfigurable wireless transceivers that automatically tune their communication parameters to the requirements of the network, resulting in improved context awareness. Nevertheless, SDR requires substantial power usage in comparison to conventional devices because of the greater complexity inherent to the sophisticated new functionalities. In this scenario, the design of energy-efficient CR sensor networks is a key challenge for the intelligent use of battery power.

Recent cognitive radio studies are interested in the development of cross-layer approaches for MAC [48,49,50] and routing [51,52]. The field of clustering protocols have been especially active. For example, the work in [53] surveys 12 papers until 2017 that use clustering topologies in CR sensor networks with a focus on energy consumption. Several subsequent works also use CR together with clustering protocols. In [54] the Learning Automata-based Multilevel Heterogeneous Routing (LA-MHR) scheme for WSNs is proposed, and [55] presents the novel approach to integrate the sensor nodes with CR nodes to forward data towards the sink using opportunistically licensed channels.

### 2.2. Data Reduction

It is clear that another way to save energy is to reduce the volume of data to be transmitted to the sink. Several methods can be used for data reduction: aggregation, compression and prediction.

It is known that the data collected at the sensors contains spatial correlation, especially in some types of measurements when the sensors are close together. If these redundant data are sent to the BS, then it causes a waste of bandwidth and an increase in the energy consumed by the nodes.

Given that the spatial correlation is stronger among data from nodes located close to each other, Heinzelman et al. [56] proposed the Low Energy Adaptive Clustering Hierarchy (LEACH) as organization of the network that consists of grouping neighboring nodes in clusters, one of them acting as Cluster Head (CH). The nodes within the cluster send data to the CH, eventually after local processing at the node. Then, correlated data received from these nodes can be processed for data reduction at the CH before being routed towards the BS. In Section 2.4.1, we address hierarchical routing and LEACH in more detail.

#### 2.2.1. Data Aggregation

The main idea under data aggregation techniques is to remove redundancies in the received data from the neighboring nodes, extracting the useful information by means of aggregation functions (maximum, minimum, average, etc.) before forwarding the final data to the BS, CH or any central node [57].

Several data aggregation schemes have been proposed that, according to the aggregation methodology, can be classified [58] in: (a) centralized (all nodes send data to an energy-rich central node, responsible for aggregation); (b) in-network (aggregation is performed in all the intermediate nodes); (c) tree-based (a minimum spanning tree is built, where root node acts as base station, source nodes are the leaves that send the data to their parent intermediate node) and (d) cluster-based (CH is responsible for aggregating the data received from the cluster nodes and then sending the data to the base station).

Data aggregation techniques is one of the most researched areas for energy efficiency in WSNs. It is noticeable that only in the last two years several surveys can be found in the literature in its field [59,60,61,62].

When sensitive data must be aggregated and transmitted, assuring privacy is also an important issue. With this focus on mind, the work in [63] surveys various existing solutions for secure data aggregation, classifying them based on the node topology and mechanisms employed for ensuring privacy.

Another interesting approach for aggregating data is to exploit machine learning to select efficient cluster heads or for dimensionality reduction of the data at sensor nodes or cluster heads. The recent survey in [64] focuses on the application of machine learning for energy efficiency in WSNs, and devotes a section to data aggregation.

#### 2.2.2. Data Compression

Another approach to reduce the amount of data to transmit is to compress these data by minimizing the number of bits required to represent each data block. All the compression algorithms are based on taking advantage of the correlation of collected data. This correlation can be temporal, among the sensed data in each node, or spatial, among the data collected in the neighboring nodes, typically inside a cluster. According to these two types of correlation, data compression mechanisms can be classified in two categories: local compression and distributed compression. In sparse sensor networks, local data compression is a good approach, but in dense networks, distributed data compression should be used [65].

**Distributed Data** **Compression** (also called in-network compression) is an asymmetric coding that uses the spatial correlation among the sensor nodes. In this case, the sensor nodes belonging to an area encode its data before sending it to the BS (or CH if clustering is used), where all the correlated flows are decoded jointly.Images and videos are usually compressed using a transform-based compression such as cosine transform or wavelets transform, where a sparse representation of the data is used to recover the original data at the decoding point with minimal loss. However, distributed transform coding (DTC) is difficult to implement in WSNs because they often need the knowledge of all measurements in the network at each node. Therefore, most articles in this field aim to modify DTC algorithms in order to make them suitable to WSNs.
  This problem can be avoided by means of distributed source coding (DSC) [66,67], based on the Slepian-Wolf theorem, since it does not require inter-communication among sensor nodes. The main problem of DSC is that it requires previous knowledge of the correlations in the data, and so its performance depends on specific assumptions.
  In contrast, compressive sensing (or compressed sensing) techniques, a relatively new idea in the field of WSNs, do not need any previous knowledge or assumption on data correlations. In compressed sensing (CS) [68] a small number of samples of a sparse signal contains enough information to successfully recover the original signal with almost no data loss. The great advantages in terms of limitations and data reduction (part of the redundant data is never acquired) make CS the most widely used technique presently in WSNs and IoT [69], even in the case of multimedia sensor networks [70].
**Local Data** **Compression** has not the limitations mentioned above and it is a universal and robust compression technique. Huffman coding, LZW (Lempel-Ziv-Welch) and RLE (Run-length encoding) are common compression techniques used in WSN for local data compression. Moreover, it can be used in conjunction with distributed data compression in WSNs to exploit both temporal and spatial data correlations.

There are several surveys about data compression techniques for energy efficiency in WSNs [71,72]. The survey in [73] focuses on in-network compression techniques, devoting special attention to CS, which is undoubtedly the most active research topic in this area in recent years. In addition, within CS schemes, the focus is currently on its use in conjunction with clustering, where interesting works have been published [74,75,76,77,78,79,80]. More recently we must mention the works in [81,82,83].

#### 2.2.3. Data Prediction

An alternative approach to save energy is to predict part of the sensed data without any transmission. Many different prediction-based data reduction mechanisms are proposed in the literature, also using different prediction approaches such as regression, neural networks, or machine learning. In [84] we can find a useful survey that also contains a systematic procedure for selecting a suitable scheme to make predictions. This work classifies the mechanisms in two groups:

1. In the Single Prediction Schemes (SPSs), predictions are made in a single point in the network, either in the cluster head (CH), or in a sensor node when it is more expensive (in energy terms) to obtain a sample than to predict one. This scheme has been applied in conjunction with adaptive sampling [85], topology control [86] and clustering [87,88,89].
2. In Dual Prediction Schemes (DPSs), where clustering is used, the predictions are simultaneously made in CHs and nodes. The idea is that nodes and CH obtain the same prediction, but the node can check the precision of the prediction by comparing this with the real measure, and only send the measure to CH if the prediction is poor. In other case, CH uses the prediction.

DPSs are potential candidates to optimize the data transmissions because they avoid unnecessary transmissions without affecting the quality of their measurements. Simulation results in [90] show that the number of transmissions can be reduced by almost 98% in the nodes with the highest load.

Wu et al. [91] propose the combined use of Dual prediction (DP) and Data Compression (DC) schemes in clustered networks, where DP is used for data reduction between nodes and CH by exploiting the temporal correlation and DC is used between the CHs and the BS by exploiting the spatial correlation.

This combination has received special attention in the literature. Jarwan et al. [92] present a comparative analysis using combinations of different mechanisms of each type. For the DP scheme, the work focuses on neural networks to perform predictions, in comparison to popular LMS approaches. Regarding data compression, principal component analysis, non-negative matrix factorization, truncated-singular value decomposition, and discrete wavelet are discussed and compared. The results show significant reduction in the number of transmissions when using both schemes while preserving the QoS requirements. More recently, the work in [93] proposes a gradient-based adaptive model that also uses a combination of DP and DC schemes.

### 2.3. Sleep/Wake-Up Schemes

Communications in WSN accounts for most of the energy consumed by network nodes, quite higher than that used for sensing and preprocessing the data they must send. Therefore, designing energy-efficient (EE) communication protocols (MAC and routing) is key to the proper functioning of the underlying application.

In any case, to choose/design the right energy-aware communication protocols, other factors such as topology and the functioning application itself must be taken into account. For instance, in WSNs deployed for critical missions (perimeter surveillance, tsunami/earthquake early detection, fire control, etc.) data must be transmitted immediately, so sensors with new information should have the highest priority to access the channel, avoiding whichever delay and contention to do so. Same way, the limited transmission range and the distance to the sink determine if a sensor must also function as a relay to help others or must lean on other itself.

#### 2.3.1. Energy-Efficient MAC Protocols

Due to its position in the protocol stack, MAC entity is ultimately the responsible for introducing and extracting packets in/from the wireless medium; and so, it is the one who commands the radio transceiver, which works in half-duplex regimen switching between transmitting and receiving modes as commanded.

From the point of view of energy efficiency, MAC protocols for WSN must minimize wasting energy in processes such as idle listening (checking actively packet arrivals), overhearing (receiving packets not intended for this node), collisions (frames lost caused by overlapping transmissions), overtransmitting (transmitting when receiver is not ready) and protocol overhead (transmitting/receiving protocol control packets/bytes). In Energy-Harvesting (EH) aware MAC protocols; however, some of these basic principles may be dynamically relaxed to achieve some kind of QoS if the external energy contribution allows it.

Almost by consensus, WSN MAC protocols are classified as Contention Free (TDMA/ FDMA/CDMA based), Contention-Based (Synchronous and Asynchronous) and Hybrid schemes (CDMA/TDMA + CSMA). However, from the point of view of energy efficiency the key concepts are Duty-cycling and Wake-up Radios.

#### 2.3.2. Duty-Cycling

Contrary to transmission, which is a synchronous activity always fired by a previous event (timer expiration, physical threshold exceeded, packet arrival), reception requires being listening in the channel prematurely (idle listening), wasting as much energy as would be wasted in any of the other two productive states (tx/rx) [3]. So being actively waiting the reception of a packet for a long time would deplete the battery very quickly, and it is not an option. In duty-cycling schemes the radio is switched off to save energy, and is only turned on periodically—during a limited interval—to check if traffic is pending somewhere in the network for this device.

Depending on the considered solution, packets ready for transmission would either make the transceiver abandon immediately the sleep state or wait for the next on period (duty cycle) to try to send them. To achieve the mandatory rendezvous between transmitter and receiver, as both must be awake simultaneously to exchange information, a wide variety of solutions have been proposed over time, and have been mostly classified as contention-based asynchronous protocols and scheduled-based with contention protocols. In [94] the reader can find a recent and updated review of duty-cycle-based MAC protocols gathering the most significative contributions till 2017, including some especially tuned for energy harvesting.

In asynchronous protocols duty cycles of different nodes are not synchronized avoiding the technical issues that will imply so precise timing adjustment, and so they are a good option for non-clustered implementations. In these schemes, rendezvous between both ends of the link is the most significant challenge. Transmitter Initiated (TI) and Receiver Initiated (RI) strategies determine a particular intrinsic subclassification, depending on which end triggers the events, with RI alternatives being more energy-efficient. Idle listening and overhearing are their main drawbacks, and though partially mitigated still remain. Nevertheless, the wide range of deployments/applications where synchronization is not only difficult but impossible, maintains the interest of the research community on them.

Scheduled-based duty-cycled protocols synchronize the beginning of the active periods of the neighboring nodes, with a not negligible cost in protocol overhead and so in energy expenditure. Internally, a leading broadcast beacon precedes a flexible virtual time structure headed by a contention period (CP) where transmitters try to multiplex their packets or organize transmission turns (reservation) in an optional glued contention free period (CFP) that would be so dynamically managed.

In wireless MAC contention protocols, the lack of a centralized access control mechanism makes collisions an issue to consider, both from the point of view of energy efficiency (useless transmissions) and QoS degradation (higher latency and wasted bandwidth). Backoff intervals are used to try to avoid collisions and to resolve those that have taken place. This has been used in several academic proposals to favor the most depleted devices (raising their priority with shorter backoff intervals) to try to avoid their collision or to shorten their contention resolution stage. CSMA/CA—and its many variants—usually with RTS/CTS packet exchange to avoid hidden terminal issue, is the regular protocol in CP intervals (both in asynchronous and synchronous-based MAC alternatives); and its Network Allocation Vector functionality (NAV) can be used by nodes in CP to tune even more their on and off intervals when a node loses contention or is not the destination of the current transmission.

Pure TDMA protocols are usually classified outside duty-cycling schemes, though really they are just a special case of contention free scheduled rendezvous where synchronized devices switch from sleep to active state following a fixed—and statically configured in advance—slot pattern, in a repetitive superframe structure. It is the best solution for those applications—industrial, critical mission, WBAN—that need deterministic (guaranteed delay and throughput) and reliable communications. Collisions, overhearing and overtransmissions are completely eliminated, and receivers, looking just in the individually assigned slots, minimize idle listening intervals. Network throughput is maximized if traffic load is high; otherwise, bandwidth is wasted as many slots will be empty but reserved. Hybrid schemes (TDMA-based with some kind of contention) look for implementing dynamic TDMA reservation, or some kind of adaptive behavior, to prevent nodes from waking up in preassigned but finally not used slots, and to adjust the contents (ownership) and length (periodicity) of the superframe structure.

Needless to say, that synchronized schemes (TDMA-based or scheduled with CP) fit perfectly in clustered architectures were the base station or virtual cluster heads can facilitate to surrounding nodes the necessary stringent time reference. If such special entities are not line powered, its special role will compromise their batteries quicker, so MAC protocols also must contribute with some kind of toggle mechanism to balance this extra consumption.

Pursuing the ideal where the receivers do not wake-up when there are no transmissions ready for them, and attending to other certainties such as that wake-up points should not be scheduled/placed without considering the characteristics of real network traffic—which can be heterogeneous and unpredictable—or that higher traffic load and smaller delay would benefit from longer duty cycles, the research community continues squeezing MAC protocol internals looking for a better duty cycle and rendezvous tuning. Following, in a very descriptive way, we introduce some of the recent contributions in the immense universe of EE (Energy-Efficient)/EH (Energy-Harvesting) WSN MAC protocols:

1. In ADP-MAC (Adaptive and Dynamic Polling-based MAC), Siddiqui et al. [95] propose modifying the polling interval distribution at the receivers dynamically, every $T_{A}$, based on the analysis of the coefficient of variation of the incoming traffic; and they resolve that the best results are achieved when the polling interval distribution is the same as that of interarrivals.
2. In AWR-PS-MAC (Adaptive wake-up interval to enhance Receiver-based Preamble Sampling MAC) [96], the transmitter reports on its particular traffic to the receiver—using piggybacking in DATA packets—and with such information the receiver adjust the next time to wake-up for this particular sender, which takes it from the corresponding ACK.
3. In ADMC-MAC (Adaptive MAC for Critical Missions) [97], a synchronous contention-based scheme, the authors design a protocol to adjust dynamically the duty-cycle of neighboring nodes, using a discrete set of values (40%, 20%, 10%), so adapting the cycle to traffic load: in a first stage, neighboring nodes determine which one will be the cluster head (each node broadcasts sync packets with the length of its transmission queue, the number of neighbors detected and its remaining energy, being such order what determines the priority in the election). Next, the selected node, using a single expression—determined after applying a regression technique to data obtained by a simulation study of S_MAC [98], its parent protocol—determines a duty-cycle-factor (a real number), which is then transformed—using some thresholds—in a duty-cycle value of a configured discrete set. Finally, this duty-cycle is broadcasted to the neighborhood and adopted as such.
4. In AP-MAC [99], to reduce the probability of collision among receiver awake intervals in a RI asynchronous scheme, the receiver add a random value to their precalculated next wake-up time before broadcasting it—via beacon frames—to the neighborhood, so transmitters can forecast the next potential rendezvous.
5. In [100], to minimize the latency in a multihop sink rooted tree topology, where data gathering is commanded by a request starting at then sink, Monica et al. [100] design a request-response protocol with extra wakeups generated dynamically to match the predicted arrival time of the response packet, as if the participating nodes were “hit” by (upward and downward) “waves”, and so sequentially awoken according to their depths.
6. In [101], the concept of duty-cycle is eliminated as a repetitive sequence of on-off periods, and is substituted by a slotted vision of time, where, self-adaptively, each node autonomously decides in every slot to sleep or wake-up. The authors claim that this way the trade off between energy-saving and packet delivery delay can be avoided. Unlike other prediction-based approaches where nodes must exchange information between each other, these enables nodes to approximate their neighbors’ situation without requesting information from them, thus saving the large amount of energy usually used for information exchange. In addition, to accomplish it, they propose an alternative approach based on game theory and a reinforcement learning technique (Q learning algorithm), where through trial-and-error interactions within the dynamic environment nodes are able to learn optimal actions.
7. Liu et al. [102] present QTSAC (Quorum Time Slot Adaptive Condensing-based MAC protocol), derived from previous QMAC family protocols [103], for achieving delay minimization and energy efficiency in a synchronized sink driven and rooted topology. As a particular rendezvous scheme, QTSAC condenses the Quorum Time Slots (QTS) into the period in which nodes transmit data—at the beginning of the polling cycle for the furthest nodes, and towards the end for those closest to the sink—which increases the number of intersection slots, improving network performance (shorter delay to meet a node in the right next-hop-group), and uses more QTS in the area that is far from the sink (according to their less or null energy expenditure acting as a relay for others).

With energy-harvesting onboard, energy efficiency constraints become relaxed someway, so the asymmetric weights of sleep/awake periods—traditionally set looking only at the remaining energy of the nodes—may now take into consideration other issues related to the provision of the QoS that certain application demands. We are mainly talking about minimizing or guaranteeing end-to-end latency, paying differentiated attention to data with different priorities, or, to a lesser extent for most WSN applications, achieving a minimum throughput. Wireless Body Area Networks (WBAN) is a good example: beyond its special environment (with possible temporal fading produced by the individual’s own movements, very close distances between sensors and sink, and moving scene), QoS is essential as sensed signals (ECG, EMG, EEG, glucose, blood pressure, SpO2) have clearly different importance and transmission requirements (latency, throughput and reliability).

8. Liu et al. [104] propose a QoS driven EE TDMA-based protocol that dynamically adjusts the transmission order and transmission duration of the nodes—frame slots reordering—and so their on/off scheduling, based on data priority, channel status (temporal fading of parts of the body) and application context (the individual’s activity, an emergency, etc.). To reduce the synchronization overhead their proposal uses guard time intervals (to deal with clock drifts), and clock tuning using data/ack exchange (piggybacking) with the personal server (onboard sink).
9. In the same research field (WBAN), Rismanian Yazdi et al. [105] design ECTP-MAC (Energy Consumption Traffic Prioritization MAC) and modify the frame structure of IEEE 802.15.4 to include an extra phase for emergency data. They define three types of data (normal discontinuous, periodic continuous and emergency) and schedule transmissions in CP, CFP, and extra interval according to a priority value proportional to data type and inversely proportional to data length and frequency.
10. In a more general context, in QPPD MAC (QoS MAC Protocol for Prioritized Data) [106], after broadcasting a beacon with QoS mode on (asynchronous contention-based RI protocol), the receiver uses a limited interval and listen for senders’ requests. The protocol implements a request-allocation scheme based on transmitter beacons and data priority tags: the highest priority transmitter beacon received at interval expiration, or, immediately, if received one tagged with the top level priority, determines which contender will obtain receiver assignment. From the point of view of EE/EH, the protocol adjusts the receiver duty cycle according to its current power level, working at 5% if remaining energy is below 10%, at full activity if battery state is above 90%, and with the value determined by a particular expression (continuous function) otherwise. The authors say the biggest advantage of their proposal over others (TDMA -based) is the energy savings due to the lack of synchronization.
11. In EEQ-MAC (Energy-Efficient and QoS-aware MAC) [107], queue length and data priority are used to adapt the node’s duty cycle, increasing the length of its active period in the event of high traffic which provides less waiting time to support time-bounded delivery of priority packets. A Random Early Detection mechanism is also included to avoid starvation of low priority data.

#### 2.3.3. Wake-Up Radios

Recently, low-power wake-up radio technology (WuR) appeared as a promising alternative, perhaps making duty-cycling unnecessary [108]. In general, duty-cycling saves energy at the cost of an increase in the end-to-end packet delivery delay. Moreover, such savings will not be optimal because of collateral effects as, for instance, protocol overhead in synchronized approaches and higher contention—so collisions and retransmissions—in collective awakenings. Finally, idle listening is not completely removed.

To achieve higher energy efficiency without increasing latency, wake-up radios—a second radio onboard—have been proposed as a promising alternative for asynchronous protocols. A wake-up radio is a low-power hardware device—consuming three orders of magnitude less power than a main RF chip [108]—capable of immediately reacting to an external event, waking up the node—and so its main radio—that is in sleep mode. Moreover with recent advancement in micro-electronics it is even possible to perform destination address decoding without waking the microcontroller, which would avoid false wakeups and overhearing [109]. It is not vain to say the only disadvantage of WuR systems appears to be the extra hardware expenditure. Though, well, limited range is another one.

Wake-up MAC protocols can be seen as on demand MAC protocols where sending nodes ask receivers to wake-up for attending an imminent packet arrival. The wake-up signal can be sent on an exclusive control channel or on the same used by the main radio; and, in the basic implementation, not even CCA is performed. In [110], state-of-the-art wake-up MAC protocols have been split into three categories that use different hardware technology: (i) duty-cycled wake-up MAC protocols, (ii) non-cycled wake-up MAC protocols, (iii) path reservation wake-up MAC protocols. The first group is a special case where duty cycling is applied to the wake-up radio, which has similar characteristics in terms of coverage range and power consumption as the main transceiver (actually something less but not enough). So neither it is attractive from the point of view of energy efficiency nor eliminates the extra delay associated with duty cycling technique.

In the second group (non-cycled) we find two interesting alternatives: those based on an always-on low-power wake-up radio (active wake-up radio) that is able of transmitting and receiving wake-up messages, and those where the wake-up radio is passive (powered by the wake-up signal, reminding RFID technology) or low-power active (using just a few micro watts from the battery), being able to receive the wake-up signal but delegating on main radio its transmission. Both share high responsiveness; active wake-up radio has greater coverage—20 m vs. ≪10 m—though leaving it active all the time conducts to a non-negligible power consumption; and passive devices achieve the highest energy conservation. Finally, in path reservation wake-up MAC protocols nodes take advantage of the additional (wake-up) radio to perform at the same time the early warning reception and a wake-up message transmission to prepare a forwarding event in a multihop context, so eliminating path establishment waiting time. Other interesting taxonomies, a hardware review, and WuR-based MAC protocol survey can be found in [111].

The clear advantage of RI schemes in duty-cycling asynchronous protocols disappears (RI vs. TI) in WuR, and, once more, the application and the scenario will determine the most suitable operation mode. In general, RI suits better for data collection whereas TI is more appropriate for event-triggered data reporting in WSNs. In the next lines we present the very last contributions in the field of EE/EH-MAC protocols:

1. In [112], FAWR (Fully Asynchronous Wake-up Radio-based MAC), a RI sink commanded scheme, is presented as a multihop WuR MAC able to overcome the short-range limitation (20 m) of this low power transceivers. Though sensor nodes are settled at one-hop distance from the BS (from the point of view of the main radio transmissions) they succeed to propagate WuR signals using a forwarding decision table built in an early topology discovery phase executed by the sink. This way, the covered area can be much larger. They also use sensor nodes’ state of charge information so that the BTS, knowledgeable about the application requirements, can properly poll deployed devices.
2. In RI-LD-WuR (RI Low Delay MAC), Singh and Sikdar [113] propose partitioning sensor nodes in k (almost) equal sized groups to reduce packet collisions in a broadcast-based RI WuR enabled WSN: the sink commands its cluster broadcasting a request in slot0—dynamic TDMA with contention—firing the data collection phase. N transmissions fit in each slot and only the sensor nodes of the respective group are able to contend for sending. They also contribute with a distributed algorithm for the initial composition of the groups.
3. In RI-CPT-WuR (RI Consecutive Packet Transmission MAC) [114], to cancel out the time wasted in collisions, the protocol enables transmitting multiple packets through a single competition.
4. In addition, precisely to avoid collisions, refs. [114,115] present different strategies to face such issue in typical asynchronous TI WuR implementations: The proposal of Guntupalli et al. [114] uses an early backoff at time to send the WuR signal, arguing that in the case of success the transmitter will not need any additional backoff to use the main radio. Ghose et al. [115], on the contrary, advocate to incorporate to the WuC (wake-up channel) a more complete MAC protocol using CCA (CCA-WuR), CSMA (CSMA-WuR) or both adaptively (ADP-WuR). In the last, a threshold determines switching between CCA and CSMA modes if CCA fails such number of consecutive attempts.
5. Finally, in SNW-MAC (Star Network WuRx MAC) [116], an Energy Management (EM) scheme is incorporated which uses the node’s residual energy to optimize the energy usage in truly EH-WSN scenarios. The protocol assumes that the time is divided into time slots of equal duration T, and the EM is executed at the end of each slot to set the throughput of the node for the next interval. First, the Energy Budget Computation module evaluates the energy that the node can consume in the next time slot k to remain sustainable; and then Throughput Computation module calculates the wake-up interval TWI[k] according to the energy budget eB[k], so determining the frequency at which the node performs sensing and sends the so-obtained data.

#### 2.3.4. Topology Control

When sensors are redundantly distributed to provide good coverage, it is possible to turn off some nodes while maintaining network operation and connectivity. Topology control protocols take advantage of redundancy to dynamically adapt the network topology based on application needs to minimize the number of active nodes. In fact, nodes that are not needed to ensure coverage or connectivity can be powered down to prolong the life of the network.

This area of research has been maintaining a moderate (tens of works per year) but continuous activity during the last ten years. In 2013 two surveys on topology control techniques were published. Li et al. [117] provide an overview of topology control techniques, classifying them into two categories: network coverage issues (blanket, barrier and sweep coverage) and network connectivity issues (power management and power control). The survey of Aziz et al. [118] presents a comprehensive study of topology control techniques for extending the lifetime of WSNs, classifying them according to the energy conservation approach they adopt.

Singla and Munjal [119] published recently a new review of topology control algorithms found in the literature, classifying them into two categories (centralized and distributed algorithms).

Next, we comment the most relevant research works published in recent years in topology control. Javadi et al. [120] propose the topology control protocol LBLATC, where every sensor has a learning automaton that chooses the most suitable transmission range using the reinforcement signal produced by neighboring nodes. As other typical problems related to WSNs, topology control can be also object of applying computational intelligence techniques. Primeau et al. [121] review the application of several computational intelligence methodologies based on fuzzy systems, neural networks, evolutionary computation, swarm intelligence, etc., to several problems in WSNs, and one of the addressed problems is topology control. Song et al. [122] propose a multihop topology control algorithm with double CH based on affinity propagation clustering (APDC-M). Exploiting the fact that combining topology control and network coding has more advantages than if we apply them separately, Khalily-Dermany et al. [123] propose a topology control algorithm where in addition to the transmission power, the consumed energy for reception is also considered. Khalily-Dermany and Nadjafi-Arani [124] also study an optimization and graph theory approach to propose a mathematical perspective for combining topology control with network coding.

### 2.4. Energy-Efficient Routing

In the network layer, the most important task in the WSN is to set up a route between sensor nodes and BS. Since routing is an additional energy-consuming task, especially in the nodes close to the sink because of forwarding much more traffic, we must use energy-aware routing protocols. For an extensive review, the most cited surveys are [9,125,126,127,128,129]. In the last two years, we can also find many surveys [130].

The survey done by Maratha and Gupta [131] (as Ogundile and Alfa [132] had done before in [132]) classifies energy-efficient routing protocols according to the communication mode towards BS into single-hop or multihop, both cases with and without clustering. In the case of clustering, the mode refers to the communication between CH and BS. In the other hand, multihop routing protocols are classified between single-path or multipath (if traffic from source to BS is balanced between several paths).

The survey by Mittal and Iwendi [133] uses a taxonomy of routing protocols based on the network structure, classifying them into flat network (or data-centric), hierarchical and location-based routing protocols. Ketshabetswe et al. [134] uses the same classification, but the QoS-aware routing protocols are also considered.

**Figure 2:**
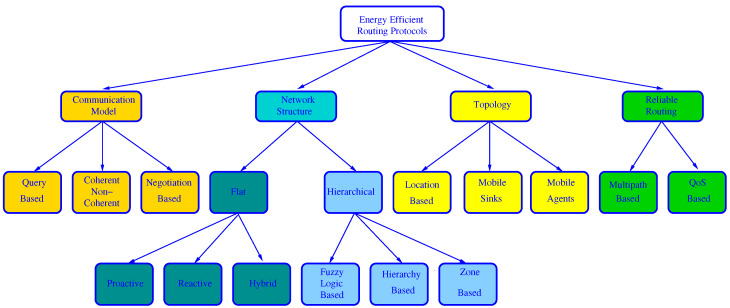
Classification of Energy-Efficient Routing Protocols used in [135].

Nakas et al. [135] made the most complete survey about EE routing because, in addition to the traditional classification category of network structure, they also consider the communication model, if location information is either used or not, and if QoS or multipath are or not supported, and moreover protocols are both described and compared in every category. This classification is shown in Figure 2.

Regarding the communication model, the protocols can be:

- Query-based: When BS needs new data, it broadcasts a query message to ask for these data. Next, the node which owns the requested data sends them to the BS.
- Coherent or non-coherent: In coherent protocols, a node applies some processing to the collected data. However, in non-coherent algorithms, the collected data are preprocessed at the source nodes and then sent to a special node, called aggregator (usually the CH), where they are further processed for data reduction.
- Negotiation-based: before real data transmission, negotiation messages are exchanged between a source node and their destination to prevent redundant data. These protocols use a naming scheme to advertise data to destination.

Regarding whether or not location information is used, this latter survey considers the special case of using a mobile agent (a program that travels across the nodes to perform tasks based on environmental conditions in an autonomous way) and where the BS can move within the network to collect data from sensor nodes. Location-based routing is also included under this type, but it could be included under the network structure category as done in other surveys.

Under the Reliable Routing category, protocols are classified depending upon their inclusion of QoS support or multiple paths to balance the load and tolerate path failures.

Another recent work [130] makes a systematic review of energy efficient routing schemes examining the literature during 2016–2018. For this purpose, they consider different categories of protocols: location-based, heterogeneity, mobility-based, multipath, hierarchical, data-centric and QoS based.

To sum up, most of the routing algorithms proposed in the literature can be classified in flat network, hierarchical and location-based, either using single-hop or multihop communication.

In flat network routing, all nodes have the same responsibility and every node has all information, so that the user can send a query to any node to obtain information. We must note that it is not possible to use a global addressing scheme due to the huge number of nodes in the network. This fact makes classical IP-based routing inadequate. Therefore, the routing is data-centric, i.e., it is totally dependent on naming of desired data.

The first data-centric protocol was SPIN (Sensor Protocol for Information via Negotiation) [136]. When a node has new data to share, it advertises this fact by transmitting an ADV message containing metadata (that identifies data). When the neighboring nodes receive this ADV message, if not repeated, they send a REQ message to the source node requesting complete sensor data. After receiving data, the process is repeated in the second node. This protocol is based on negotiation, but data-centric algorithms are mainly query-based, as is the case with Direct Diffusion [137] and Rumor Routing [138].

In location-based routing, all the nodes calculate the distances to the neighboring nodes based on incoming signal strength. Another option is to use a GPS signal, but in this case, nodes should go to sleep mode whenever the communication is not active. Some major routing protocols of this type are Geographic Adaptive Fidelity (GAF) [139] or SPAN [140], that is integrated with IEEE 802.11 to improve transmission latency, and extend the network lifetime.

As already explained in Section 2.2, since in most WSN applications data flows from sensor nodes to BS, and data from nearby nodes contains redundancy, clustering favors data reduction by exploiting the spatial correlation. This is the basis of hierarchical routing protocols, where nodes are grouped in clusters, and a node is selected as the CH, that is responsible for collecting data from the sensor nodes inside the cluster and routing it towards the sink, directly (single-hop) or through several CHs (multihop). Hierarchical protocols are the most popular and the preferred option for WSNs and they are clearly the most studied protocols presently. For this reason, we will focus on them.

#### 2.4.1. Hierarchical Routing Protocols

**Figure 3:**
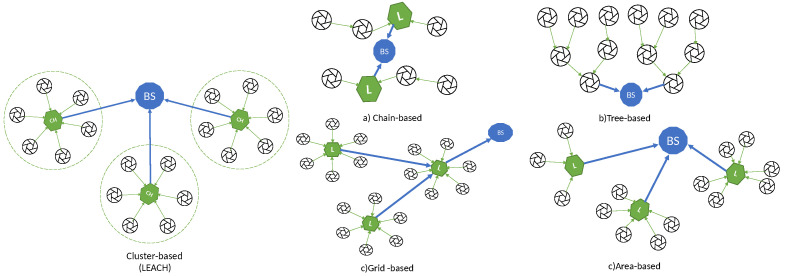
Different hierarchical routing strategies (BS base station, L leader, CH cluster head).

Heinzelman et al. [56] proposed the first hierarchical routing protocol referred to as LEACH (Low Energy Adaptive Clustering Hierarchy). LEACH proposes a random rotation method to select the node with maximum energy level as the CH, and so uniformly distribute the energy load among the sensors in the network. CHs send advertisement messages to the whole WSN using CSMA. Each sensor node joins the cluster from which it receives the strongest signal. Next, CH schedules TDMA slots for each member in the cluster to send data to it. CH uses aggregation techniques to combine the data received from sensor nodes to save energy and bandwidth, and then this aggregated information is forwarded directly to the BS, i.e., using only one hop, as shown in Figure 3.

The single-hop transmission is the simplest method, but usually it is not suitable for large networks, where multiple-hop transmission should be employed. In this case, data follows a multiple-hop route across several CHs towards the BS, and so it is essential to use an energy-aware routing protocol that avoids unnecessary transmissions and the overload in the nodes close to the BS.

Clustering enhances energy efficiency in several ways: (i) it reduces the communication range within the cluster and so less transmission power is necessary, (ii) data reduction techniques can be performed by the CH, (iii) energy-intensive operations such as coordination or data reduction are only carried out by the CH, (iv) it enables the powering-off of some nodes, typically after sending data to the CH. On the other hand, hierarchical routing also improves network scalability by maintaining a hierarchical topology in the network.

LEACH is still the most important and most used basic routing algorithm for WSNs. After 18 years of existence, much attention is still devoted to LEACH by the research community working in the area of routing in WSN. This itself shows its relevancy. In several recent works [141,142,143], the authors survey, classify and analyze different versions or improvements of LEACH, also using multihop transmission.

Manjeshwar and Agrawal [144] proposed another popular cluster-based routing algorithm referred to as Threshold-sensitive Energy-Efficient sensor Network (TEEN) [144] that has been designed for time critical applications. TEEN combines the architecture based on clustering with the use of a data-centric mechanism. Adaptive Periodic TEEN (APTEEN) [145] is an enhancement of TEEN where CH broadcasts relevant parameters to the cluster members such as threshold values, TDMA schedule, and maximum time between consecutive reports.

Another interesting cluster-based routing protocol is Hybrid Energy-Efficient Distributed (HEED) [146], where CH election is triggered in given intervals and it is based mainly on residual energy and other parameters as the number of neighbors or the distance to them. A survey recently published by Ullah [147] focus on HEED-based protocols.

Since the relevancy of cluster-based routing, it is common to speak indistinctly of cluster-based and hierarchical routing, but strictly speaking, other types of hierarchical structures have been proposed in the literature. Recently, Chan et al. [148] survey and compare both LEACH-based clustering and these other hierarchical structures, classified into the following categories: (a) chain-based, (b) tree-based, (c) grid-based, and (d) area-based, also represented in the Figure 3.

In chain-based hierarchical routing, the WSN is divided into chains; and a leader is chosen for every chain. Every node sends the data to the next node until the leader is reached. The main drawbacks are the delays suffered by the farthest nodes in long chains, the overload of the nodes close to the leader and the connectivity loss in a sub-chain when a node fails. The most relevant chain-based algorithm is PEGASIS (Power-Efficient Gathering in Sensor Information Systems) [149], where the leaders are rotated for energy reasons, and they send the aggregated data to the sink.

In tree-based routing algorithms, a sink tree is created and there is a single path between each node and the sink. Unlike the chain-based case, there are no leaders, and a parent node can receive data from several children (or leaves), unlike the previous case, a node (parent) can have several children that send data to it, enabling the possibility of aggregation. The main drawbacks are similar to the chain-based case, i.e., the delays suffered by the farthest nodes in long trees, the overload of the nodes close to the sink and the connectivity loss in branches connected to a parent that fails. The most relevant tree-based algorithm is PEDAP (Power-Efficient Data gathering and Aggregation Protocol) [150] that uses the optimum sink tree based on data volume and transmission distance.

In grid-based algorithms, the whole network is split into many grids (similar to clusters), based on the geographical location of the nodes. The leader selected for every grid is the responsible for routing the data through other leaders until reaching the sink, i.e., using multihop transmission. Each node only needs to know the location information about the leader of the grid. The most important proposal of this type is Two-tier data dissemination (TTDD) [151], where the mobile sink use flooding to send a data request to source nodes.

In area-based mechanisms, the entire network is divided into multiple variable-sized areas. The BS also transmits a data request to the closest nodes that they forward via flooding until the data source is reached, which will send the data to the sink. A typical area-based algorithm is Line-based data dissemination (LBDD) [152], where a line of leaders is selected to split the whole network in two areas. The nodes send data to the closest leader on the line, and the leaders on the line store data from nodes and serve requests from sink if possible, and if not, send the request up and down the line. A little improvement was proposed in Ring Routing [153], using a ring instead of a line.

**Figure 4:**
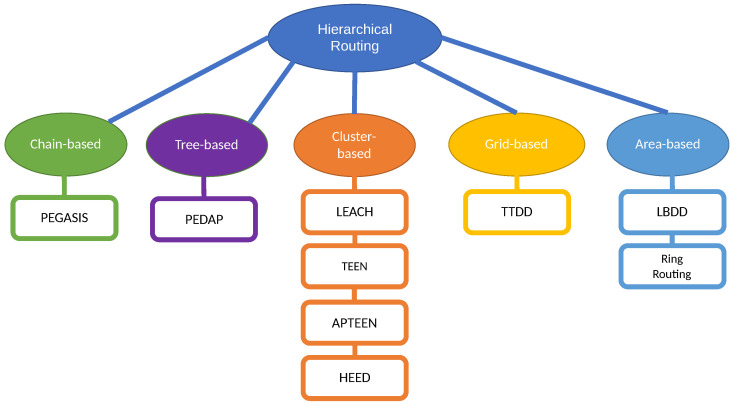
Classification of hierarchical routing strategies.

In Figure 4, we show the most relevant algorithms of each type.

The main problems related to cluster-based routing are the cluster formation, the selection of CH in each cluster and the relay node placement. In addition to the classic approaches to address them, these problems have been object of optimization in hundreds of works in last years, and they are clearly one of the most active research areas presently. To solve this problem, the researchers have appealed to optimization (Swarm Intelligence Algorithms) and methodological approaches such as fuzzy logic or metaheuristic. The survey in [154] studies hierarchical energy-efficient routing protocols based on classical and swarm intelligence approaches.

However, the subsequent survey in [155] focuses on methodological approaches in cluster-based multihop routing protocols that are classified into four categories: classical approaches, fuzzy-based approaches, metaheuristic-based approaches and hybrid metaheuristic- and fuzzy-based approaches.

**Figure 5:**
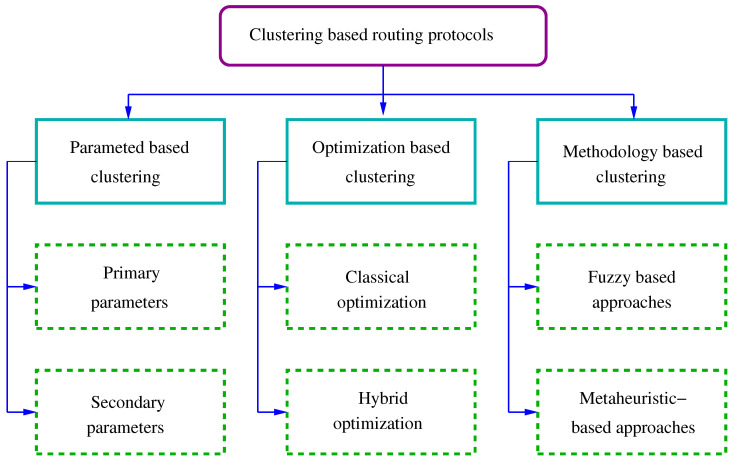
Classification of clustering-based routing protocols reproduced from Manuel et al. [156].

The very recent survey by Manuel et al. [156] is much more complete and the classification considers both swarm intelligence algorithms: ant colony optimization (ACO), artificial bee colony optimization (ABCO), fuzzy logic (FL), genetic algorithm (GA), whale algorithm (WA), or particle swarm optimization (PSO) and methodological approaches (fuzzy-based and metaheuristic-based approaches). See Figure 5 reproduced from this complete survey.

A review of the recent literature on these topics shows rapidly the high level of research activity in this area. The work in [157] studies the adoption of sink mobility to avoid the hot spot problem (CHs close to the BS). The mobile sink moves within the network and communicate directly with CHs without the need for routing. The ACO algorithm is studied for finding an optimal trajectory for the mobile sink. In [158] a special clustering method called Energy Centers searching using PSO (EC-PSO) is proposed for clustering and CH selection. In [159] a firefly algorithm is developed for selecting the CH optimally. Ezhilarasi and Krishnaveni [160] propose the evolutionary multipath energy-efficient routing protocol (EMEER) using a cuckoo search algorithm to optimally select the CH considering energy efficiency. Recently, Alghamdi [161] proposes an optimal CH selection by considering energy, delay, distance, and security using a new algorithm that hybridizes the concept of dragon fly and firefly algorithms. Additionally, recently, the Optimized QoS-based Clustering with Multipath Routing Protocol (OQoS-CMRP) has been proposed [162] by applying the Modified Particle Swarm Optimization to form clusters and select CHs.

Ref. [163] introduces an algorithm that uses fuzzy logic for cluster construction and CH selection, and ACO for inter-cluster routing to mitigate the hot spot problem and extend network lifetime. In [164] an interesting PSO-based unequal and fault tolerant clustering protocol (PSO-UFC) is presented. In [165] the authors use a cuckoo optimization algorithm (COA) for clustering and selection of optimal CHs, considering four criteria such as the remaining energy of nodes, distance to the base station, within-cluster distances, and between cluster distances. In [166] a multihop LEACH protocol is optimized by means of an ACO algorithm, using a CH close to the BS. Other recent works that propose LEACH optimizations are the proposal in [167] using a Fuzzy C-means clustering (FCM) Algorithm, the work in [168] that uses a PSO algorithm or the optimization made in [169] by means of a Genetic Algorithm. Another interesting work is that of Jain and Goel [170] where fuzzy sets and fuzzy decision rules have been used for intelligent selection of CHs and to setup multihop routes to BS.

Although LEACH is the preferred protocol for using as basis for optimization, other cluster-based protocols are also used. Therefore, several improvements of PEGASIS has been recently proposed. In [171] an Enhanced PEGASIS (EPEGASIS) protocol is proposed to mitigate the problem of hot spots from four directions. The work in [172] combines PEGASIS with Hamilton Loop algorithm, through a mixture of single-hop and multihop mechanisms, inserting a mobile agent node that is responsible for receiving and merging packets from the CHs. The authors in [173] also combines PEGASIS with a genetic algorithm to build the chain instead of the greedy algorithm.

The problem of CH selection in APTEEN using artificial intelligence has also attracted the interest of researchers in recent years: using PSO [174], a combination of genetic algorithms and fruit fly optimization algorithm [175], or ACO [176,177].

## 3. Energy Harvesting and Energy-Neutral Operation

Sensor nodes are usually powered by a battery with limited capacity. The use of conventional batteries does not always permit designing long-lasting WSNs. Moreover, replacing batteries can be too difficult when severe environmental conditions exist. Therefore, to avoid having to replace batteries, one possibility is to recharge the battery of the nodes by means of an energy harvesting system.

Energy-harvesting techniques allow the sensor nodes to obtain energy from the external environment such as from sun, wind, vibrations, radio frequency, thermal, etc. These techniques transform the energy of the environment into electrical energy that can be used in the nodes. This energy can be used to avoid depletion of the node batteries. Energy harvesting does not guarantee that the nodes can operate continuously and indefinitely because the energy sources are uncontrollable, making them unpredictable and difficult to model. An example is sunlight, which is not available at night, or wind whose speed is variable and difficult to predict. Although sensor nodes could operate by adapting energy consumption to the energy harvested at any given time, it will often be desirable to store the harvested energy for later use. For this purpose, sensor nodes should be equipped with storage devices such as rechargeable batteries, although the use of supercapacitors has become more popular due to their high energy storage density and their smaller size, which is more suitable for WSN nodes. A fundamental problem in EH-WSNs is to properly manage both the energy consumption in the WSN and its energy-harvesting system.

In a battery-powered node, the goal of a power-management scheme is to minimize the energy consumption or to maximize the lifetime achieved while required performance constraints are satisfied. In an energy-harvesting node, one possibility is to consider the harvested energy as a supplement to the battery energy and try again to maximize the lifetime of the network. However, a more interesting possibility in this case is to use the energy at an appropriate rate so that the WSN keeps on operating continuously. This mode was called energy-neutral operation (ENO) [178]. Therefore, a node is said to obtain energy-neutral operation if the desired level of performance can be sustained indefinitely.

In this section, we are interested in the power-management mechanisms and ENO in EH-WSNs. We will refer to them as neutral power management. Moreover, we will focus on energy prediction strategies.

The research area related to power management and ENO in EH-WSNs is moderately active (one hundred papers a year indexed by Scopus), but there has been a slight increase in the last few years. However, only a couple of interesting surveys have been published in last years about power-management mechanisms and ENO in EH-WSNs.

In [179], Adu-Manu et al. present the technologies for harvesting energy from ambient sources (RF, solar, mechanical, thermal and hybrid). Moreover, it dedicates one section to the architecture of a energy-harvesting node (storage, energy prediction and power management). Finally, they study the protocols that can take advantage of the harvested energy: physical layer (power adjustment based on link quality parameters, multi antenna-based protocols or joint source-channel coding-based protocols), MAC layer (adaptive duty-cycle-based protocols, CSMA/CA-based protocols), routing (offline routing protocols, route cost-based protocols, geographic routing-based protocols and clustered network-based protocols).

The review presented by Sah and Amgoth [180] surveys and discusses several works published until 2019 that propose methods to reduce energy consumption in EH-WSNs (clustering and routing, energy balancing, coverage awareness and node placement) and essential prediction strategies for maximizing the energy harvesting of the sensor nodes (solar, wind, mechanical and thermal).

**Figure 6:**
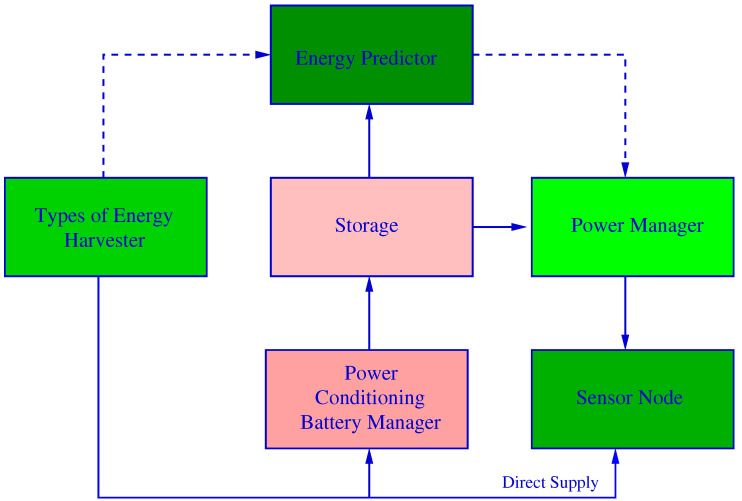
Components of a EH-WSN system. Adapted from [181].

This survey includes an illustrative figure reproduced from Basagni et al. [181], where the components of an EH-WSN system are shown (in Figure 6 we can see an overview of this EH-WSN system). The energy obtained by the harvester(s)can be used directly to cover present energy needs by the node or it can be stored for later use. When the current energy usage is lower than the harvesting rate, the excess energy can be stored in the buffer for later use, thus supporting variations in the energy harvested from the environmental source. The Power Manager is responsible for controlling the appropriate energy rate supplied from the storage to the node. Another important module is the Energy Predictor that controls the operation of the Power Manager, using the information from the energy harvesters and the energy level in the storage. Energy prediction is important because protocols can be optimized if we have knowledge from the energy that can be harvested in the short and the long term.

Next, we comment the most relevant research works published in 2019–2020 both in energy prediction and neutral power management.

### 3.1. Energy Prediction

One of the most popular energy predictors is the solar energy prediction model based on an Exponentially Weighted Moving-Average (EWMA) proposed by Kansal et al. [178], which takes into account energy harvested in the previous days. This model was improved by the prediction method Weather-Conditioned Moving Average (WCMA) proposed by Recas Piorno et al. [182].

Another popular energy prediction model is Pro-Energy (PROfile energy prediction model) by Cammarano et al. [183], based on using past observations for both sun and wind. The main idea is to use profiles representing the energy available for different types of “typical” days (for example, sunny, cloudy, or rainy). Recently, Deb and Roy [184] propose a modification of Pro-Energy but using real-life solar traces for prediction.

Sharma et al. [185] propose a new approach for energy predictions, based on weather forecasts, which is valid for sun and wind. Jankovic and Saranovac [186] present a solar energy predictor that also uses cloud cover and precipitation probability predictions from weather forecast. The errors of humidity and atmospheric pressure are used to feed the fuzzy logic filter.

In [187], Sharma and Kakkar also present a recent survey on solar energy forecasting techniques published before 2019 (time series models, neural networks and other advanced models based on machine learning, genetic algorithm or fuzzy logic). Moreover, a detailed study on power management techniques is also included (data aggregation, routing, adaptive duty cycle, among others).

Ma et al. [188] propose a Correlation Least Mean Square (C-LMS) prediction model for solar energy that introduces the correlation factor of weather changes.

Herrería-Alonso et al. [189] propose a novel energy prediction model that makes use of the altitude angle of the sun at different times of day to predict future solar energy availability. The great advantage of this interesting approach is that it does not need to maintain local energy-harvesting patterns of past days, or any particular tuning for each different scenario or location.

### 3.2. Neutral Power Management

In the scope of routing, Bahbahani and Alsusa [190] propose a cooperative clustering protocol based on LEACH to enhance the longevity of EH-WSNs. The CH role is alternated between the nodes using duty-cycling. Sah and Amgoth [191] propose a novel energy harvesting clustering protocol (NEHCP) that uses solar energy harvesting.

In the scope of MAC, Pegatoquet et al. [112] present a novel MAC protocol for EH-WSNs exploiting ultralow-power wake-up radios. Additionally, a multihop wake-up scheme based on a dual radio system is proposed to solve the problem of the limited range typical of wake-up radios. Chamanian et al. [192] propose an adaptive duty-cycling algorithm which provides ENO according to the energy available in the environment, using two different vibration-based harvesters: piezoelectric and electromagnetic.

In [193], Zhu et al. consider the problem of deploying EH directional sensor networks for optimal target coverage, involving directional sensing coverage, route selection and ENO.

## 4. Wireless Power Transfer (WPT) and Simultaneous Wireless Information and Power Transfer (SWIPT)

In the area of 5G networks, massive IoT and sensor networks the goal is to provide reliable communications under requirements of low complexity, cost and power. One promising piece of the solution to the latter requirement is energy harvesting from radio frequency signals. The original far-field Wireless Power Transfer (WPT) paradigm with dedicated frequencies to send recharging beams to devices is evolving towards harvesting the energy from radio frequency signals already there, either ambient signals or the ones used for communication as it happens with Simultaneous Wireless Information and Power Transfer (SWIPT). This evolution parallels the one from disjoint power and communication lines towards the integrated use of the former in Power Line Communications (PLC) as pointed out in [194].

In recent times several surveys have been published around WPT and SWIPT, some of them focused on some particular application.

The survey by Perera et al. [194] describes thoroughly WPT and SWIPT technologies. It covers Near-Field WPT used to recharge devices at short distances, Far-Field WPT used to transfer energy at longer distances, SWIPT architectures such as separate receivers for power and data communication, either time switching or power splitting in the shared receiver approach, and antenna switching. It also presents interference exploitation approaches in SWIPT. Finally, it comments upon several emerging scenarios amenable to a SWIPT approach such as WPT/SWIPT on cooperative relay, SWIPT-enabled cooperative non-orthogonal multiple access (NOMA) and secure WPT/SWIPT transmission.

A brief survey by Liang et al. [195] presents an overview of SWIPT, explores its application on several 5G scenarios and discuss the mmWave network case.

The survey by Hossain et al. [196] focuses on the SWIPT technology as used mainly in cooperative relay networks. Allowing a base station to transmit the power needed by a device to altruistically relay data nullifies the cost in battery power that it would incur into otherwise. This approach guarantees the collaboration of all nodes in a more efficient quid pro quo network operation.

A broader survey by Hu et al. [197] centers on use, redistribution, trading and planning of energy harvested in future wireless networks interoperating with smart grids. SWIPT technology is the focus of its seventh Section. Its Section XI identifies SWIPT as a promising technology in Smart Grid-Powered Wireless Communications applied to 5G such as NOMA-based wireless powered sensor networks and Unmanned Aerial Vehicle (UAV) aided communication networks. In Subsection XII.E it identifies several lines of future research in SWIPT Systems.

**Figure 7:**
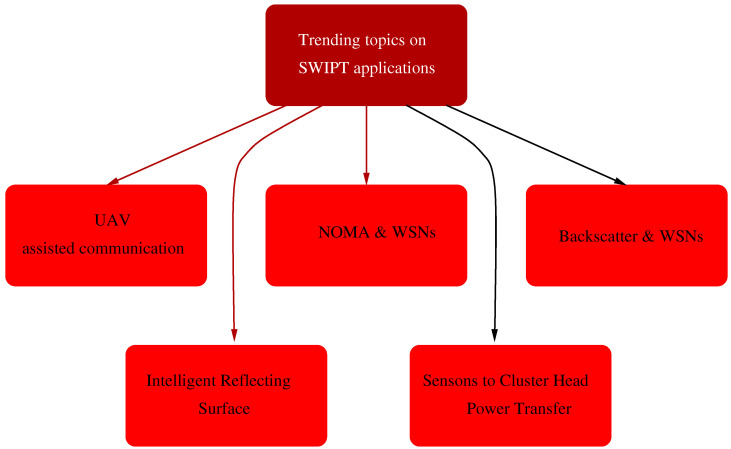
Trending topics in research about SWIPT/WPT in WSNs.

To complete the overview of SWIPT applications related to WSNs, several trending topics in recent research can be identified as shown in Figure 7.

Several active lines of research deal with UAV assisted communication networks, some of them with higher potential for the WSNs case. Hu et al. [198] focus on WPT, computing the UAV trajectory that maximizes the minimum received energy among all ground nodes. This would be a feasible approach for recharging sensor nodes in case their ambient energy-harvesting capabilities would not suffice. Huang et al. [199] center on power allocation and trajectory design of UAVs in a SWIPT-enabled IoT network. Hassan et al. [200] explore UAV deployment and clustering of nodes in case of disaster and unavailability of conventional networks. This design could be adapted to WSNs to make them more resilient. Wang et al. [201] propose a UAV-aided SWIPT-enabled NOMA scheme to guarantee secure transmission for ground passive receivers (PRs). Well-designed WSNs with single ownership should be less prone to eavesdropping and denial of service attacks though.

Wu and Zhang [202] explore the use of Intelligent Reflecting Surface (IRS) assisted SWIPT scenario with a multi-antenna Access Point (AP) and both multiple single-antenna either information or energy users. This approach would be able to save power capabilities in WSNs, especially those with some level of geographical clustering.

NOMA where several devices simultaneously transmit using the same frequency at different power levels such that receivers can reconstruct the original signals through interference cancellation brings about a more efficient use of wireless communication resources. Its use in WSNs has also been proposed. Nguyen et al. [203] evaluate a SWIPT-enabled WSN deploying NOMA and compare the performance of both time switching (TS) and power splitting (PS) approaches in terms of outage probability. Several optimization studies deal with the general case of SWIPT-enabled NOMA systems. We comment upon several of them without being exhaustive. Yuan et al. [204] propose a design aimed to maximize system wide energy efficiency of a full-duplex cell-center relaying a cell-edge user’s data. Rajaram et al. [205] propose several SWIPT schemes for cooperative communication systems with multiple relays and multiple destination users communicating simultaneously. Luo et al. [206] propose a deep learning approach in order to determine an approximation to the optimal solution for the minimization of the total transmit power of a SWIPT-enabled NOMA system while satisfying the quality of service requirements.

Choi and Lee [207] propose sensor nodes with high harvesting energies recharging the cluster head through SWIPT to maximize the achievable rate of sensing data transmission while guaranteeing ENO.

He et al. [208] analyze the influence of interference on SWIPT, propose an interference-aware route metric and design an interference-aware SWIPT routing algorithm.

Backscatter technology aims to use an ambient signal (like WiFi [209] or LTE [210]) for communication from a tag-device through passive radio using a low-power reflective RF switch and a passive antenna towards a receiver. Zheng et al. [211] propose using backscatter to enhance a Wireless Powered Communication Network collaboration between two devices that first harvest wireless energy from a Hybrid Access Point (HAP) and afterwards transmit their information to the HAP. Hence, the weaker device backscatters the received energy signal to transmit its information to the relay device in a passive manner. Tuo and Zhang [212] explore the outage probability of a relay harvesting energy through power splitting from both a primary source and a secondary backscattering one at the same time that forwards both their data. They study the performance of the hybrid system through analysis and simulation, and find a larger throughput gain than in a single primary link scenario at the cost of negligible degradation of the primary throughput.

## 5. Discussion and Future Directions

A wide range of applications as diverse as the one that underlies the concept of sensor networks, with such disparate geographical scenarios ranging from deployments with minimum distances between sensors and BS (e.g., WBAN) to those that rely on satellite transponders to receive data, with very demanding quality of service requirements in some cases (industrial environments, alarm, surveillance, e-health…), creates a scenario where multiple alternative solutions/mechanisms will coexist, not necessarily complementary/compatible, perhaps even designed ad hoc for a particular application. In addition, the possibility of energy-neutral operation (ENO) of network elements only further complicates the range of techniques that will have to be properly combined to achieve proper system operation.

The fact that we have focused on natural energy sources (sun, wind, temperature gradient…) for recharging the batteries of the sensors, and the inherent unpredictability of these sources, means that the dynamic management of the remaining energy of the device, and of that which can be collected in the near future, is an important factor in the tasks to be carried out by the onboard processor.

The incorporation into this research field of techniques for predicting energy that can be harvested is one of the hot points, and we can find from proposals based on complex automatic learning techniques to simpler solutions that try to avoid the processor’s own energy consumption in this prediction. The combination of different harvesting mechanisms in the same sensor and the related problems is also an interesting topic for further research. In this sense, the possibility of exploiting the radio frequency signals that surround us ubiquitously as a source of energy is one of the most promising issues and where there is currently a lot of potential for improvement.

When energy is harvested from radio signals, it can be done not only from ambient ones already there (WiFi, LTE) but from signals sent explicitly by dedicated equipment or some node of the network for either only power transfer (WPT) or for dual use, i.e., both information and power transfer (SWIPT).

With respect to special equipment, research on unmanned aerial vehicles mainly assisting in energy needs of the network has been even focused on disaster and unavailability situation. This research may bring about more resilient WSNs.

Special case of dedicated equipment is that of intelligent reflecting surfaces (IRS) assisting an access point in conquering higher power efficiency of its sensor set, especially with some geographical clustering of its nodes. As a mirror image scenario there is a proposal to power up the cluster head in a WSN with the surplus energy from its dependent nodes.

Several lines of research focus on NOMA networks, such as power and time splitting comparison or minimization of total transmit power in the network while abiding to service requirements.

Finally, backscatter technology can be a disrupting technology to WSNs. In mainly urban areas with high level of LTE signals they would even permit batteryless operation of sensor nodes. Other approaches consider a hybrid network with backscatter nodes and active transmitting ones collaborating towards a more efficient WSN. Finally, one of the most promising technologies in this area is that of the combination of backscattering with either an ambient EH or properly SWIPT framework, achieving dual radio signal reusing both in the power and the information domains. Only the first steps have been done towards its use in WSNs and future research may deal with different aspects such as multihop and hybrid nodes design implications.

The rise of machine learning techniques can also have a beneficial impact on achieving an energy-autonomous application. Adjusting the energy expenditure of devices (i.e., the actions they can perform at any given time) to both the energy remaining and the energy expected to be collected in the immediate future can be very useful, so incorporating predictive techniques that allow anticipating random changes in certain natural energy sources seems peremptory. In solutions where the receiver initiates the information exchanges (RI-MAC protocols), automatic learning techniques could also help the transmitter to synchronize its activation times with the receiver’s listening, avoiding energy loss while waiting for the sounding beacon. In any case, complex techniques involving excessive computation may be counterproductive (they require their own energy expenditure), or even not applicable in devices with very limited resources.

From the point of view of communication protocols, recent contributions in the field of wake-up radios allow devices to make considerable energy savings by largely avoiding idle listening and overhearing processes, although their applicability is severely restricted by the short distances over which the technique would be effective. Another promising research field is related to the study of the energy consumption associated with the cryptographic mechanisms with which we want to protect communications, given the capital importance that security has acquired presently.

On the other hand, as the mobility of network elements (e.g., intelligent transport systems) is increasingly present in our applications, which implies a greater use of radio, it will be necessary to continue redesigning routing and MAC protocols to make them more energy-efficient (using, for example, techniques such as opportunistic forwarding).

Undoubtedly, the most productive area of research for energy efficiency in WSNs is cluster-based routing, with LEACH-based protocols playing a major role. This is because in most WSN applications, data flows from sensor nodes to the BS, and data from nearby nodes contains redundancy, so clustering favors data reduction by exploiting spatial correlation. This fact has also led to a huge research activity in the field of data reduction, and more specifically in data aggregation techniques.

In the area of cluster-based routing, the new ideas based on heuristic and fuzzy approaches can allow the clustering methods to achieve more optimal results. The CH and relay nodes selection is always an open research area for cluster-based routing as it directly affects performance. In the other hand, most approaches are designed to work only for a small networks. New research work should be conducted to design more scalable clustering techniques which can work with larger and changing network sizes. Finally, while the architectural implications of different scenarios (single-hop or multihop) justify the proposal of alternative communications protocols, it may be interesting to design highly configurable (MAC/routing) protocols that could provide more than acceptable performance for applications with different needs.

In the field of data aggregation, there are two main future directions for researching. On the one hand, assuring privacy is an important issue when sensitive data must be aggregated and transmitted. Another interesting approach for aggregating data is to exploit machine learning to select efficient cluster heads or for dimensionality reduction of the data at sensor nodes or cluster heads.

Compressive sensing (or compressed sensing) is another relatively new idea for data reduction since it does not need any previous knowledge or assumption on data correlations. The great advantages in terms of limitations and data reduction (part of the redundant data is never acquired) make compressive sensing the most widely used technique presently in IoT, even in the case of multimedia sensor networks.

To conclude this discussion, we have observed that generically speaking, it is extremely difficult to make comparisons between the different alternative proposals made by the research community in the different areas related to energy efficiency in WSNs (data aggregation, compressed sensing, MAC and routing protocols, clustering-related problems such as CH and relay nodes selection…). In this sense, it would be fascinating to define a set of adequately characterized typical scenarios. In this same direction, there is a lack of empirical results that could support that the advantages of certain mechanisms—studied by means of simulation techniques—do not vanish or are feasible when implemented in a real environment, with the hardware restrictions inherent to these devices. Nor did we find any conscientious studies that consider—from the point of view of energy expenditure—such important factors as the mobility of the sensors, and the security of communications, which would increase the time of use of the radio and the processor, with the consequent impact on the life of the batteries.

## 6. Conclusions

This paper compiles the contributions of recent years (until the end of 2020) in various research fields related to sensor networks, and which address from different perspectives the problem of extending the lifetime of the underlying system/application. To this end, we classify—as some other authors did previously—the multiple techniques proposed in the literature under the headings: energy conserving, energy harvesting and energy transfer. Most of the techniques are orthogonal, i.e., they could operate together, although the heterogeneity of the scenarios may make some of them from very appropriate and interesting, to unprofitable or directly unimplementable. Within the techniques that address the conservation problem, the issues related to the transmission/reception processes are critical, since the radio is the most energy-intensive component in a sensor, whose first and last objective is to transport some information to the base station. At all levels of the communications architecture of a sensor (at physical, link, network and application level) we find contributions to minimize energy consumption, although always at the cost of some other type of performance (increased latency in delivery, lower accuracy of the data collected, etc.). The possibility of harvesting energy from natural sources represents a paradigm shift, although it is not a panacea due to their unpredictability. The use of rechargeable batteries and the dynamic management of the harvested energy bring closer not only the possibility of providing a certain quality of service to the collection of information (compliance with time constraints and/or minimum bandwidth), but also, at the limit, the achievement of an energetically self-sufficient system (ENO). In addition, in a world increasingly populated by radio frequency signals the possibility of using them as an energy transport mechanism or even energy supply, opens a very promising outlook for sensor networks.
